# The Effect of Different Types of Bariatric Surgery on Levothyroxine Requirement in Hypothyroid Bariatric Patients

**DOI:** 10.7759/cureus.26165

**Published:** 2022-06-21

**Authors:** Dickson Dewantoro, Rachel Davenport, Jing Yi Goh, Andisheh Bakhshi, Abdulmajid Ali

**Affiliations:** 1 General Surgery, University Hospital Ayr, Ayr, GBR; 2 Emergency Medicine, University Hospital Ayr, Ayr, GBR; 3 Statistics, University of West of Scotland, Glasgow, GBR; 4 General Surgery, University Hospital Ayr/University of West of Scotland, Ayr, GBR

**Keywords:** weight loss and obesity, levothyroxine, bariatric surgery, hypothyroid, effect of bariatric surgery

## Abstract

Background

Bariatric surgeries are carried out to improve a patient's quality of life, and to reduce respiratory, cardiac, endocrine, and metabolic complications encountered by those with high body mass index (BMI). A complication associated with high hypothyroidism is weight gain, which may lead to obesity. Here, we explore the effect of different bariatric procedures on thyroid function and levothyroxine dosage.

Methods

This is a retrospective review of 887 patients referred to a tertiary bariatric service between 2008 and 2020 and treated for hypothyroidism at the time of referral.

The study identified 57 patients on thyroxine replacement. Of these, 22 underwent restrictive bariatric procedures, 16 underwent restrictive/malabsorptive procedures, and 19 did not undergo operative intervention.

Comparisons were made among each group throughout the timeline of interest.

Results

Out of 57 patients, 50 (87.7%) were female. The average age for patients was 47.26+/- 8.89 years. The average BMI at baseline was 48.72+/- 8.68 kg/m2. The mean dose of levothyroxine in controls was 115.8+/- 53.5 mcg while that of surgical patients was 149.8+/- 68.4 mcg.

There were no statistically significant differences in levothyroxine doses between surgical and control at T0 (baseline), T1 (one-year post-op in surgical patient, or two-year post referral in control patient), T2 (two-year post-op in surgical patient, or three-year post referral in control patient), and T3 (most recent result available). The surgical group was then categorized further into restrictive and restrictive/malabsorptive. When they were compared with the control group, there were no statistically significant differences in doses.

A generalized linear mixed model was applied to assess differences in levothyroxine dose with time as a random variable. This was adjusted for age, sex, BMI, T4 level, and hypothyroid cause. Through this assessment, there were several statistically significant differences in levothyroxine dosage between the groups. Control group required on average 28.06 mcg less levothyroxine than the restrictive/malabsorptive group (p=0.015). Also, the restrictive group required on average 23.57 mcg less levothyroxine than the restrictive/malabsorptive group (p=0.033). There were no statistically significant differences observed between the control group and the restrictive group (p=0.67)

Conclusion

Patients who have bariatric surgery have changes to their anatomy and physiology which may affect both their thyroid hormone homeostasis and levothyroxine pharmacokinetics. Thus, hypothyroid bariatric patients requiring levothyroxine must have their thyroid function monitored regularly. In this study, it was found that hypothyroid patients who underwent restrictive surgery had an overall statistically significant lower levothyroxine requirement to remain euthyroid as compared to the restrictive/malabsorptive group (p=0.033). Additionally, the control group required statistically significantly less levothyroxine than the restrictive/malabsorptive group (p=0.015). These factors may determine the type of surgery chosen by hypothyroid bariatric patients. However, further studies that are randomized, controlled, and multi-center with a higher population are required.

## Introduction

Bariatric surgery provides various benefits to patients presenting with a high body mass index (BMI) and associated comorbidities of the respiratory, endocrine, and cardiovascular systems as well as a significant improvement in reported quality of life [1]. There are two main categories of bariatric surgery: restrictive (R) and restrictive-malabsorptive (R/M). An example of restrictive bariatric surgery is the gastric sleeve and gastric band while an example of restrictive-malabsorptive is roux-en-Y gastric bypass. Post bariatric surgery, weight loss results through multiple mechanisms and there are anatomical and physiological changes that affect the absorption of nutrients and medications.

Hypothyroidism

Due to many underlying causes, the body may not be able to produce an appropriate response to endogenous thyroxine, and patients develop hypothyroidism. The development of hypothyroidism is one of the known causes of weight gain, and patients with a high BMI are known to have an increased demand for thyroxine to maintain a euthyroid state.

Hypothyroidism is a relatively common disease, with a prevalence of 2% of the general population in the UK. Prevalence is higher in women than in men [2]. There are several causes of hypothyroidism which can be grouped into primary and secondary (central). Primary hypothyroidism causes about 99% of hypothyroidism, which are conditions affecting the thyroid gland, including autoimmune destruction, irradiation injury, and surgical partial or total thyroidectomy [3]. Hypothyroidism is managed with thyroid replacement therapy such as levothyroxine, with a narrow therapeutic range.

The starting dose of levothyroxine is determined by body weight. The titration of the dose is then managed according to response following periodical monitoring of thyroid function tests (TFTs), comprising thyroid-stimulating hormone (TSH), and thyroxine (T4) [2,4].

Bariatric surgery and hypothyroidism

In this study, we are looking at the effect of bariatric surgery on thyroid function, as demonstrated by levothyroxine dose and TFTs. Available literature demonstrates varying outcomes on the effect of bariatric surgery on the requirement for levothyroxine replacement therapy. A retrospective study in 2017 stated that bariatric surgery reduces the requirement for thyroid replacement therapy in bariatric patients with concomitant hypothyroidism one year following surgery, with 13% of patients no longer requiring any thyroid replacement [5]. Conversely, a case report published in 2020 described the development of iatrogenic hypothyroidism following gastric sleeve surgery [6].

Operative procedures

Roux-en-Y Gastric Bypass (Gastric Bypass)

A reversible procedure with the creation of a small pouch in the stomach, and a gastroenterostomy to the intestine. This enterostomy bypasses the duodenum and a variable length of jejunum, causing both reduction in the functional size of the stomach, as well as restriction of absorption of nutrients in the small intestine [7].

Sleeve Gastrectomy (Gastrectomy)

A non-reversible restrictive procedure, where the stomach is divided longitudinally, reducing the volume of the stomach, with preservation of the lower oesophageal sphincter and pylorus.

Gastric Banding

A reversible restrictive procedure whereby a constricting eccentric ring is surgically inserted around the fundus of the stomach.

Pharmacokinetics of thyroid replacement therapies

The absorption of levothyroxine mainly occurs in the small intestine, especially the jejunum and ileum [8]. In gastric sleeve procedures, and during gastric banding, there is no alteration to the length of the small bowel [9]. During gastric bypass, the length of the small bowel that remains for the absorption of nutrients depends on how distal the anastomosis has been formed [10]. The more proximal the anastomosis in bypass, there is more absorption of fats, starches, and selected minerals and fat-soluble vitamins. Length of the small bowel is also likely to affect the absorption of levothyroxine and may affect the dosage required for an adequate replacement to maintain a patient within a euthyroid level.

Gastric bypass and possibly sleeve gastrectomy have both been shown to increase the pH of the stomach, with mini-gastric bypass causing a significantly higher increment in gastric pH [11]. Despite the increase in pH, patients who had sleeve gastrectomy tend to be put on proton pump inhibitors due to acid reflux side effects. A higher gastric pH has the effect of decreasing the absorption of levothyroxine [8]. This may also increase the required dose of levothyroxine.

Bariatric surgical patients often also require the replacement of other essential minerals, including iron and calcium supplementation. Replacement of these minerals with calcium carbonate and ferrous sulfate can also affect the absorption of levothyroxine.

## Materials and methods

This is a retrospective study on the service evaluation of patients referred to the bariatric surgery team at a tertiary bariatric unit in Scotland. By interrogating the database of all patients referred for bariatric intervention, we were able to identify those patients who had been treated with thyroid replacement treatment. Of these 887 patients, 57 were identified as hypothyroid (TSH of greater than 4.2 mU/L and T4 of less than 10 pmol/L), 38 had surgical intervention, and 19 did not proceed with bariatric surgery.

Data were collected from the patient notes portal to determine who had undergone bariatric surgery and to record the dose of levothyroxine prescribed at the time of referral, at one-year post-procedure, at two years post-procedure, and the current dose of thyroid replacement. Where available, the patient’s weight and BMI were recorded for each of the above-mentioned timeframes, along with their TFT results. The type of procedure carried out, the age of the patient at referral, and the patient’s gender were also recorded.

For patients who had not proceeded with bariatric surgery, the dates for data collection were at the time of referral, at two years post referral, three years post referral, and current. This timeframe was selected as patients undergoing surgery waited on average one year for the procedure to be carried out. This group acted as a control group, as they comprised patients who were of a similar weight and comorbidity profile to those in the surgical group and fulfilled the same criteria, including age between 18 to 60; BMI of 35 kg/m2 or more; and type 2 diabetes mellitus or sleep apnoea.

The findings were then compared initially between the control group and those who underwent surgery. Further comparison within the surgical group is further categorized as either restrictive or restrictive/malabsorptive. Statistical analysis was performed with the help of a departmental statistician through the generalized linear mixed model approach.

## Results

Baseline

Data for 57 hypothyroid bariatric patients, who were diagnosed with hypothyroid before referral to bariatric service, were included in this study, of which 50 (87.7%) were female. The average age at baseline was 47.26 ± 8.89 years old while the average BMI at baseline was 49.72 ± 8.68 kg/m2.

There were four main causes of hypothyroidism observed. Forty-nine (86%) patients had a diagnosis of autoimmune hypothyroidism; three (5.3%) patients were hypothyroid following radioiodine treatment; one (1.7%) patient with subtotal thyroidectomy; and four (7%) had a total thyroidectomy. All on average were taking levothyroxine with a dose of 132.46 ± 64.43 mcg.

Baseline TSH showed there were 19 (33.3%) patients with abnormally high values (normal value of TSH being 0.27-4.2 mU/L), 23 (40.4%) patients with normal values, seven (12.3%) patients with abnormally low value and eight (14%) patients with no available TSH.

In terms of T4 at baseline, there were four (7%) patients with abnormally high values, 44 (77.2%) patients with normal values, one (1.8%) patient with abnormally low values, and 8 (14%) patients with no available T4 value.

Out of the 57 patients included, 19 (33.3%) did not receive any surgical intervention and thus, acted as a control group. Of those who received surgical intervention, 22 (38.6%) patients underwent restrictive bariatric procedures while 16 (28.1%) patients underwent a restrictive/malabsorptive procedure.

At baseline, there were no significant differences in patients’ age, BMI, levothyroxine dose, sex, T4, and cause of hypothyroidism among the three study groups (no surgery, restrictive, and restrictive/malabsorptive). This allowed us to observe the difference over time among the different groups. The TSH was inversely proportional to T4. The findings at baseline are summarised in Table 1

**Table 1:** Descriptive statistics at baseline (referral) BMI: Body mass index; TSH: Thyroid-stimulating hormone *. The mean difference is significant at the .05 level.

		No Surgery (n=19)	Restrictive (Band, Sleeve, Balloon) (n=22)	Malabsorptive (Bypass) (n=16)	Total (n=57)	p-value
Age		46.11 (10.65)	46.91 (8.99)	49.13 (6.34)	47.26 (8.89)	0.597
BMI		50.57 (9.94)	49.71 (8.52)	48.73 (7.68)	49.72 (8.68)	0.827
Levothyroxine Dose		100 (100)	137.5 (81)	137.5 (100)	132.46 (64.43)	0.323
Sex	Female	16 (84.2%)	20 (90.9%)	14 (87.50%)	50 (87.7%)	0.88
	Male	3 (15.8%)	2 (9.1%)	2 (12.50%)	7 (12.3%)	
TSH	High	12 (63.2%)	4 (18.2%)	3 (18.8%)	19 (33.3%)	0.026*
	Normal	6 (31.6%)	9 (40.9%)	8 (50%)	23 (40.4%)	
	Low	0 (0%)	4 (18.2%)	3 (18.8%)	7 (12.3%)	
	NA	1 (5.3%)	5 (22.7%)	2 (12.5%)	8 (14%)	
T4	High	0 (0%)	3 (13.6%)	1 (6.3%)	4 (7%)	0.164
	Normal	18 (94.7%)	13 (59.1%)	13 (81.3%)	44 (77.2%)	
	Low	0 (0%)	1 (4.5%)	0 (0%)	1 (1.8%)	
	NA	1 (5.3%)	5 (22.7%)	2 (12.5%)	8 (14%)	
Hypothyroidism Cause	Autoimmune	17 (89.5%)	19 (86.4%)	13 (81.3%)	49 (86.0%)	0.697
	Radioiodine	1 (5.3%)	1 (4.5%)	1 (6.3%)	3 (5.3%)	
	Subtotal Thyroidectomy	1 (5.3%)	0 (0%)	0 (0%)	1 (1.8%)	
	Total Thyroidectomy	0 (0%)	2 (9.1%)	2 (12.5%)	4 (7.0%)	

Levothyroxine dose in control group vs surgical group

When the levothyroxine dose in the control group was compared to the combined surgical group, several findings were observed.

At T0 (baseline), there was no significant difference in levothyroxine dose (p=0.169). The mean levothyroxine dose for the control group was 115.8 ±53.5 mcg while that of the surgical group was 140.8 ±68.4 mcg.

**Figure 1:**
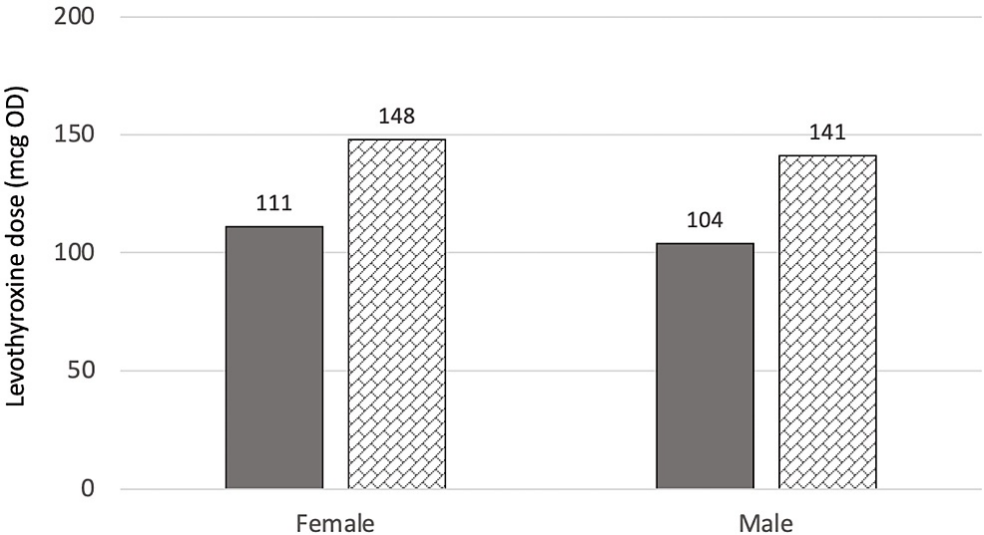
Estimated marginal means of dose at T1 in relation to sex mcg: Microgram, OD: Once daily

**Figure 2:**
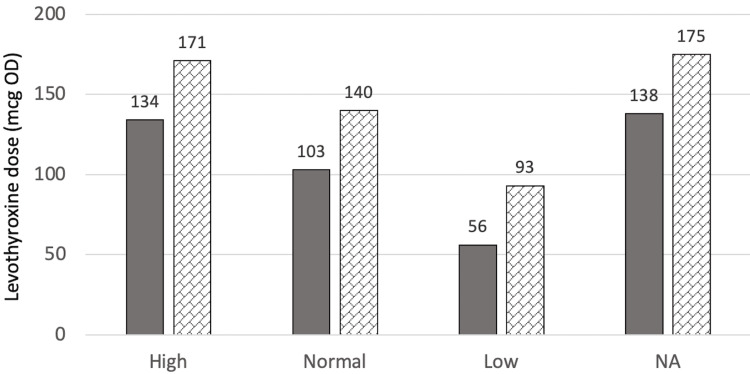
Estimated marginal means of dose at T1 in relation to the T4 category mcg: Microgram, OD: Once daily, NA: Not available

At T1 (one year after surgery for the surgical group or two years after referral for the control group), there were no significant differences in levothyroxine dose between the two groups (p=0.339). However, the surgical group was estimated to require a higher levothyroxine dose (p=0.167) at 141.5±71.0 mcg when adjusted for age, BMI at T1, sex, and T4 value at T1 as compared to the control group with a mean dose of 123.7±52.4 mcg. While this might be important medically, it is not statistically significant. Figure 1 and Figure 2 show the estimated marginal means of dose at T1 when compared to sex, and T4 categories, respectively.

**Figure 3:**
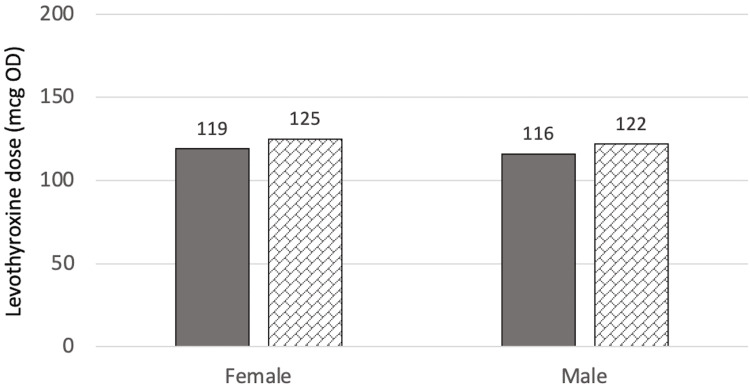
Estimated marginal means of dose at T2 in relation to sex mcg: Microgram, OD: Once daily

**Figure 4:**
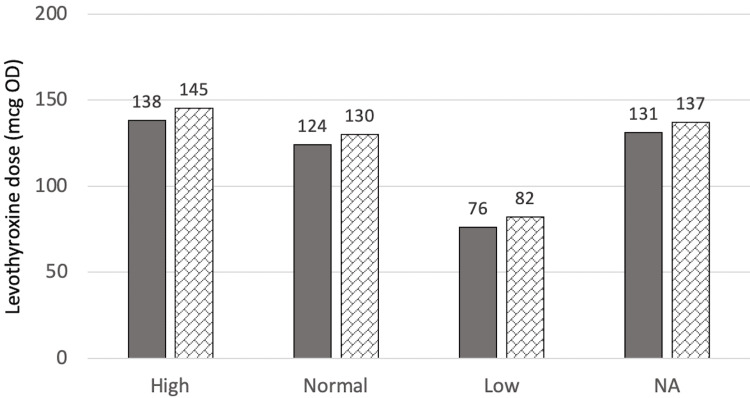
Estimated marginal means of dose at T2 in relation to T4 category mcg: Microgram, OD: Once daily, NA: Not available

At T2 (two years after surgery for the surgical group or three years after referral for the control group), there were no significant differences in T2 levothyroxine dose between the two groups (p=0.748) even when adjusted for age, BMI at T2, sex, and T4 at T2 (p=0.855). The mean dose for the control group was 123.7±52.4 mcg and for the surgical group was 143.3±69.9 mcg. Figure 3 and Figure 4 show the estimated marginal means of dose at T1 when compared to sex, and T4 categories, respectively.

**Figure 5:**
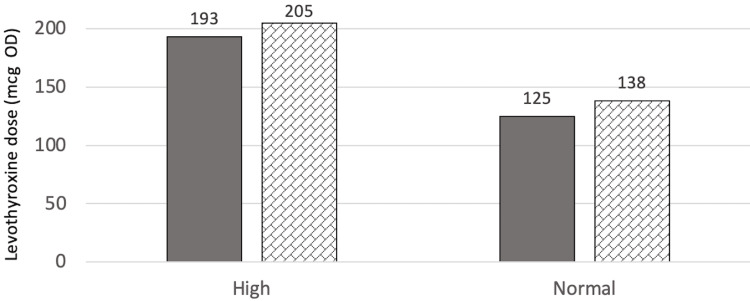
Estimated marginal means of dose at T3 in relation to T4 categories mcg: Microgram, OD: Once daily

**Figure 6:**
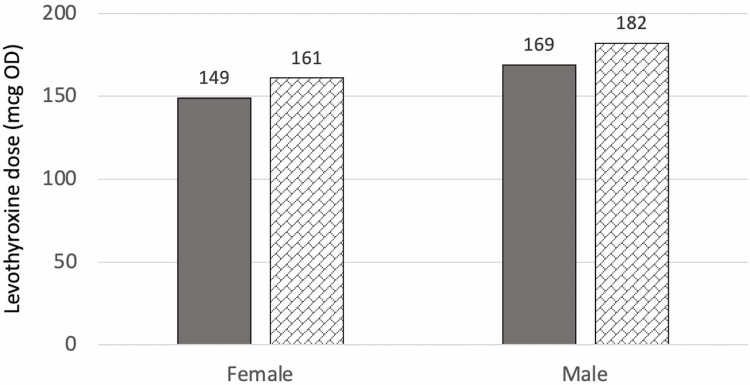
Estimated marginal means of dose at T3 in relation to sex mcg: Microgram, OD: Once daily

At T3 (the most recent result available), there were still no significant differences in levothyroxine dose between the control group with a mean dose of 127.6±64.5 mcg, and the surgical group with a mean dose of 133.6±64.0 mcg, (p=0.744) even when adjusted for age, BMI at T3, sex, and T4 at T3 (p=0.619). Figure 5 and Figure 6 show the estimated marginal means of dose at T3 when compared to sex, and T4 categories, respectively.

Overall, in T1, T2, and T3, there are higher estimated marginal means of levothyroxine dose in the surgical group as compared to the control group. The difference in levothyroxine dose, while medically significant, is not statistically significant.

Levothyroxine dose in control group vs restrictive group vs restrictive/malabsorptive group

In this analysis, the surgical group was further categorized into a restrictive group and a restrictive/malabsorptive group. All three groups were compared against each other.

When comparing all three groups at T0, there was no significant difference in levothyroxine dosage (p=0.323). The mean dose for control, restrictive, and restrictive-malabsorptive groups are 115.8±53.5 mcg, 135.2±73.9 mcg, and 148.4± 61.6 mcg, respectively.

**Figure 7:**
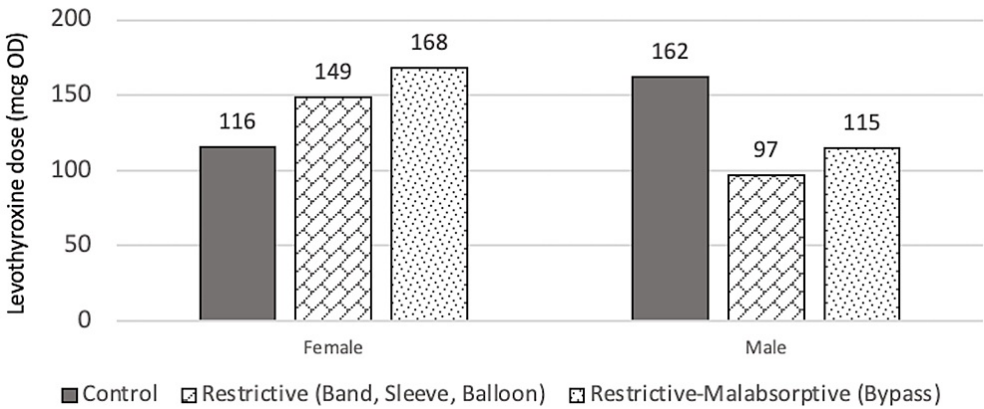
Estimated marginal means of dose at T1 in relation to sex mcg: Microgram, OD: Once daily

**Figure 8:**
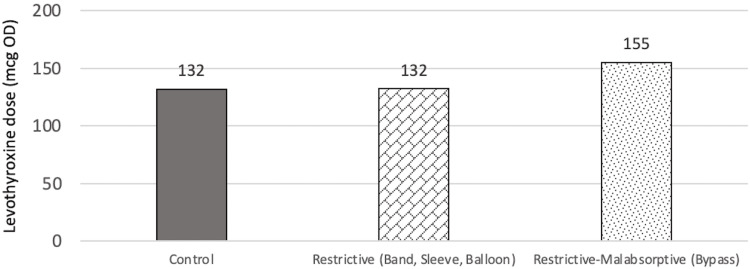
Estimated marginal means of dose of each group at T1 mcg: Microgram, OD: Once daily

At T1, there was no significant difference in levothyroxine dose between all three groups (p=0.363), even when adjusted for age, BMI at T1, sex, and T4 value at T1. The mean dose for control, restrictive, and restrictive-malabsorptive groups are 123.7±52.4 mcg, 131.8±75.7 mcg, and 154.7±64.0 mcg, respectively. It can be observed through the model that there was a higher levothyroxine dose requirement for males than for females in the control group, and the opposite pattern in both the restrictive and restrictive/malabsorptive group. It can also be observed that patients within the restrictive/malabsorptive group require the highest levothyroxine dose at T1. However, these findings may have low statistical reliability due to very few numbers of males in the study and high variation among individuals. The findings at T1 are summarized in Figure 7 and Figure 8.

**Figure 9:**
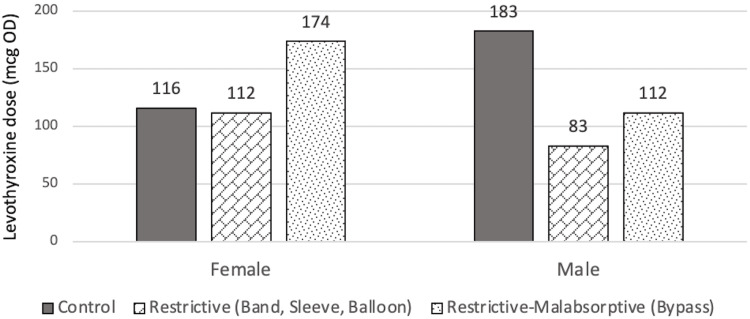
Estimated marginal means of dose at T2 in relation to sex mcg: Microgram, OD: Once daily

**Figure 10:**
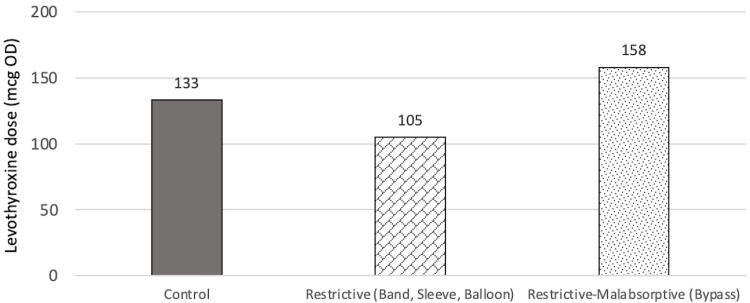
Estimated marginal means of dose of each group at T2 mcg: Microgram, OD: Once daily

Levothyroxine dose findings at T2 showed similar findings as in T1. There were no statistically significant differences observed (p=0.382). The mean dose for control, restrictive, and restrictive-malabsorptive are 130.0±63.5 mcg, 123.8±76.8 mcg, and 157.1±68.9 mcg, respectively. The relationships of doses at T2 are summarized in Figure 9 and Figure 10.

**Figure 11:**
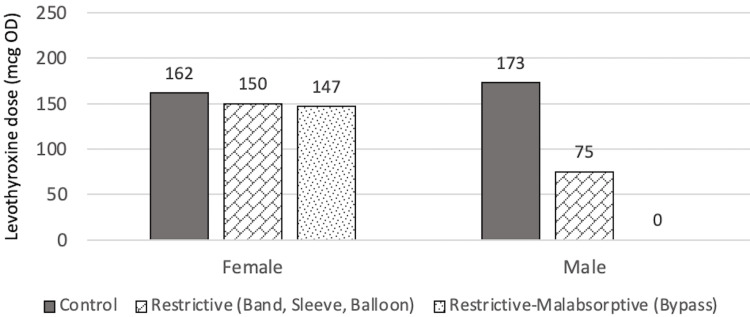
Estimated marginal means of dose at T3 in relation to sex mcg: Microgram, OD: Once daily

**Figure 12:**
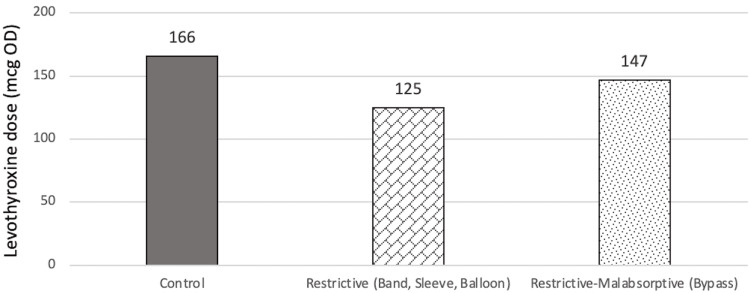
Estimated marginal means of dose of each group at T3 mcg: Microgram, OD: Once daily

At T3, there were no observed statistically significant differences in levothyroxine dose among the three groups (p=0.453), similar to the findings at T1 and T2. The mean dose for control, restrictive, and restrictive-malabsorptive groups are 127.6±64.5 mcg, 122.7±66.3 mcg, and 148.4±59.5 mcg, respectively. The finding at T3 is summarized in Figure 11 and Figure 12.

Levothyroxine dose with time as a random variable

Further assessment was done through the generalized linear mixed model (GLM) approach where time was used as a random variable and group as a fixed factor. This model was also adjusted for sex, age, BMI, T4 level, and cause of hypothyroidism.

This model shows several statistically significant levothyroxine dose differences among groups. Control group required on average 28.06 mcg less levothyroxine than the restrictive/malabsorptive group (p=0.015). Also, the restrictive group required on average 23.57 mcg less levothyroxine than the restrictive/malabsorptive group (p=0.033). There were no statistical significances observed between the control group and the restrictive group in need of levothyroxine (p=0.67). This comparison is described in Table 2.

**Table 2:** Generalized linear mixed model: Multiple group comparison Based on observed means. The error term is Mean Square (Error) = 4357.595 *. The mean difference is significant at the .05 level Multiple comparisons — dependent variable: Levothyroxine dose Std. Error: Standard error; Sig.: Significant

(I) Surgery Type II	(J) Surgery Type II	Mean Difference (I-J)	Std. Error	Sig.	95% Confidence Interval for the difference	
					Lower Bound	Upper Bound
Control	Restrictive (Band, Sleeve, Balloon)	-4.49	10.517	.670	-25.22	16.24
	Restrictive-Malabsorptive (Bypass)	-28.06^*^	11.437	.015*	-50.60	-5.51
Restrictive (Band, Sleeve, Balloon)	Control	4.49	10.517	.670	-16.24	25.22
	Restrictive-Malabsorptive (Bypass)	-23.57^*^	10.971	.033*	-45.20	-1.94
Restrictive-Malabsorptive (Bypass)	Control	28.06^*^	11.437	.015*	5.51	50.60
	Restrictive (Band, Sleeve, Balloon)	23.57^*^	10.971	.033*	1.94	45.20

**Figure 13:**
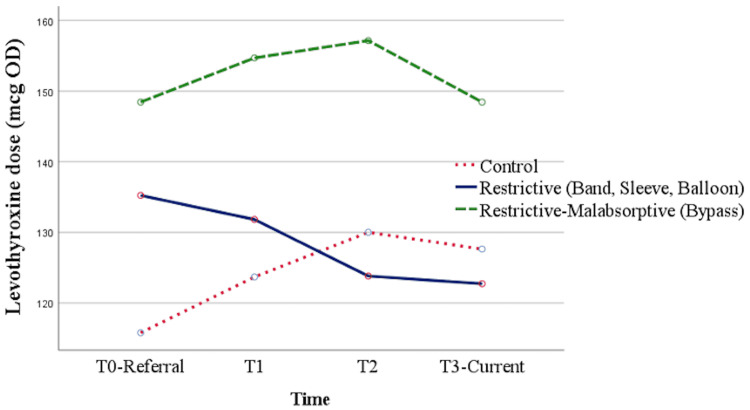
Estimated mean figures of dose over time by the study group mcg: Microgram, OD: Once daily

Overall, through the GLM model approach, the mean levothyroxine dose requirement was highest in the restrictive/malabsorptive group throughout T0, T1, T2, and T3. Meanwhile, as time progresses from T0 to T3, initially, there was a higher average dose requirement for the restrictive group as compared to the control group. This stayed true at T1, albeit the differences were smaller. By T2, the control group required a higher levothyroxine dose than the restrictive group and this remained true until T3. The difference between the control and restrictive group, as mentioned before, were not statistically significant. Figure 13 shows the differences in the average levothyroxine dose in each study group throughout the studied timeline according to the GLM model approach.

There were patients for whom data in the form of blood results were not available within the time frames of the study. When comparing and assessing normal, high, low, and unknown (NA) T4 level categories, it was found that the differences in the levothyroxine dose between NA T4 and other categories are significant. This suggested that it might be medically relevant as to who these patients with NA T4 levels were. We also found that in all significant cases, patients with NA T4 have a higher levothyroxine dose. The statistically significant differences were between those with low T4 levels and the NA category (p=0.035) and between normal T4 levels and NA T4 levels (0.006). If these results were available, then there might have been a significant effect on the results of the study. The comparisons among T4 level categories are summarised in Table 3

**Table 3:** Generalised linear mixed model: Multiple T4 level categories comparison Based on estimated marginal means *. The mean difference is significant at the .05 level. a. An estimate of the modified population marginal mean (I) b. An estimate of the modified population marginal mean (J) d. Adjustment for multiple comparisons: Least significant difference (equivalent to no adjustments) Pairwise comparisons — dependent variable: Levothyroxine dose Std. Error: Standard error; Sig.: Significant; NA: Not available

(I) T4 Category	(J) T4 Category	Mean Difference (I-J)	Std. Error	Sig.^d^	95% Confidence Interval for Difference^d^	
					Lower Bound	Upper Bound
High	Low	85.582^a,b^	61.459	.173	-39.185	210.350
	NA	-38.486^a,b^	36.418	.298	-112.419	35.446
	Normal	35.177^a,b^	31.368	.270	-28.504	98.857
Low	High	-85.582^a,b^	61.459	.173	-210.350	39.185
	NA	-124.069^a,b,*^	56.553	.035*	-238.878	-9.259
	Normal	-50.406^a,b^	53.967	.357	-159.965	59.153
NA	High	38.486^a,b^	36.418	.298	-35.446	112.419
	Low	124.069^a,b,*^	56.553	.035*	9.259	238.878
	Normal	73.663^a,b,*^	24.994	.006*	22.922	124.404
Normal	High	-35.177^a,b^	31.368	.270	-98.857	28.504
	Low	50.406^a,b^	53.967	.357	-59.153	159.965
	NA	-73.663^a,b,*^	24.994	.006*	-124.404	-22.922

Through the GLM model approach, the influence of the cause of hypothyroidism on levothyroxine dose was also studied. In several cases, the cause of hypothyroidism has a statistically significant influence on the levothyroxine dose. Patients within the total thyroidectomy group, on average, required 78.797 mcg, 108.496 mcg, and 171.834 mcg more levothyroxine than those in the autoimmune group (p=0.014), radioiodine group (p= 0.014) and subtotal thyroidectomy group (p=0.006), respectively. These pairwise comparisons are described in Table 4.

**Table 4:** Generalised Linear Mixed Model: Multiple Hypothyroidism Cause Comparison Based on estimated marginal means *. The mean difference is significant at the .05 level a. An estimate of the modified population marginal mean (I) b. An estimate of the modified population marginal mean (J) d. Adjustment for multiple comparisons: Least significant difference (equivalent to no adjustments) Pairwise comparisons — dependent variable: Levothyroxine dose Std. Error: Standard error; Sig.: Significant

(I) Hypothyroidism Cause	(J) Hypothyroidism Cause	Mean Difference (I-J)	Std. Error	Sig.^d^	95% Confidence Interval for Difference^d^	
					Lower Bound	Upper Bound
Total Thyroidectomy	Autoimmune	78.797^a,b,*^	30.403	.014*	17.076	140.519
	Radioiodine	108.496^a,b,*^	41.788	.014*	23.663	193.329
	Subtotal Thyroidectomy	171.834^a,b,*^	59.318	.006*	51.412	292.256

Lastly, pairwise comparisons were performed between sex groups. Through this assessment, there was no statistical significance found in levothyroxine dosage. The mean difference between females and males was 22.028 mcg (p=0.36). This is described in Table 5. The comparatively small number of male patients referred to the bariatric service who was also hypothyroid affected the power of this comparison.

**Table 5:** Generalised linear mixed model: Sex group comparison Based on estimated marginal means a. An estimate of the modified population marginal mean (I) b. An estimate of the modified population marginal mean (J) c. Adjustment for multiple comparisons: Least significant difference (equivalent to no adjustments) Pairwise comparisons — dependent variable: Levothyroxine dose Std. Error: Standard error; Sig.: Significant

(I) Sex	(J) Sex	Mean Difference (I-J)	Std. Error	Sig.^c^	95% Confidence Interval for Difference^c^	
					Lower Bound	Upper Bound
Female	Male	22.028^a,b^	23.735	.360	-26.157	70.213

## Discussion

Control vs surgery

There are contrasting forces affecting the trends of thyroid function and levothyroxine dose in bariatric patients. Levothyroxine absorption is affected by many factors including gastric pH, gastric volume, and length of the small intestine [8,12]. Concurrent medications can also affect the absorption of levothyroxine, particularly proton pump inhibitors, ferrous sulfate, and calcium carbonate, which are commonly prescribed in patients who have undergone a bariatric procedure [8]. However, the homeostasis of thyroid hormones is also affected by total body weight, which is planned to decrease following bariatric surgery. The required dosage for replacement may therefore be affected to differing extents by the different bariatric operations.

We compared the effects on thyroid function and levothyroxine dosage in patients who underwent bariatric surgery in comparison to a group of patients who had also been referred to the bariatric surgery team, but who had not proceeded with operative intervention. There appeared to be an improvement in thyroid function over time, although this was not a significant finding, as indicated by a reduction in levothyroxine dose in patients who proceeded with surgery, while there was an increase in the dose of levothyroxine in patients who were in our control group. Notably, patients in the surgical group had a higher average dose of levothyroxine at all stages of T0, T1, and T2.

A 2018 study of 90 American bariatric patients showed that both sleeve gastrectomy and gastric bypass improved thyroid function, and were associated with a reduction in thyroid replacement therapy, and also that this reduction was independent of weight loss [13].

Another study of thyroid function and BMI, in the context of bariatric surgery, noted a marked and significant reduction of thyroid replacement therapy dose, and reduction in BMI one year after the procedure in both bypass and gastric sleeve patients [5]. This includes discontinuation of levothyroxine in 13.2% of patients. This was theorized to be related to a better distribution of levothyroxine due to a reduction in excess body fat.

None of the patients in our study were found to have discontinued levothyroxine, and there were no significant trends in levothyroxine dose.

A study into patients with subclinical hypothyroidism, who had received gastric bypass, a restrictive/malabsorptive surgery found that there was a reduction in the TSH in all patients, but that it did not correlate to BMI [14].

Restrictive vs control

Patients undergoing restrictive surgery showed a decrease in the required dose of levothyroxine. This was reduced below the average dose seen in the control group. The dose required by the restrictive/malabsorptive group remained higher than that of the control group. This was not however a statistically significant result. Differences in dose between the two groups may represent a change in the pharmacokinetics of the medication due to bypass of the small intestine and the dramatic reduction in stomach size and length of the continuous small intestine.

Pharmacokinetics of levothyroxine has been shown to play a role in other studies, including a case report describing a patient presenting with recurrent features of hypothyroidism following sleeve gastrectomy [6]. The TSH in this patient continued to rise, despite increasing the levothyroxine dose. Symptom resolution and TFTs only resolved once patients had begun to crush the medication instead of swallowing it whole. Stomach pH was felt to play a role here.

In our setup and many others, patients who had an operative intervention are followed up closely by the bariatric service, with regular appointments with the surgical, pharmacy, and nursing teams. This may well represent those patients who attend the pre-operative assessments and who attend follow-up. These patients may be more likely to have their blood monitored, and have in the past been found to be more compliant with medication [5].

Limitations and recommendations

There are several limitations related to this study that should be considered when undertaking further studies. Firstly, the retrospective nature of this study meant that we were unable to control patients’ exposure to environmental and other medical factors. To tackle this issue, a randomized controlled trial should be performed.

The study was single-centered, and therefore, it does not represent the overall population. Additionally, the high variation of components studied associated with a relatively small study sample, means the observations made from the study cannot be generalized and that results could differ in a different sample population. Therefore, further collaborative multi-site studies should be done to improve the generalization of the results.

The much lower proportion of male hypothyroid bariatric patients as compared to female hypothyroid bariatric patients affected the power of the comparison. However, this represents the fact that hypothyroidism is more prevalent in females than males [15]. This was confirmed by a study that was performed in Norway, which showed a female-to-male ratio of 5.4 [16]. Nevertheless, more population numbers are required to increase statistical significance.

## Conclusions

In summary, patients who have bariatric surgery have changes to their anatomy and physiology which may affect both their thyroid hormone homeostasis and the pharmacokinetics of levothyroxine. Through these mechanisms, we may expect the dose required to remain euthyroid to change. In this study, we have been unable to show whether the factors decreasing dose requirement outweigh those favoring a decrease in dose. It is essential that regardless of the surgery performed, each patient requiring thyroid hormone replacement has their thyroid function monitored regularly after their bariatric surgery to ensure they are neither over nor under-treated given the narrow therapeutic range and the consequences of falling outside this range. While we observed that hypothyroid patients who underwent restrictive surgery had an overall lower requirement of levothyroxine to remain euthyroid compared to the restrictive/malabsorptive group, this finding was statistically insignificant. Meanwhile, the statistically significant findings include that the control group required less levothyroxine than the restrictive/malabsorptive group. Additionally, the restrictive group required less levothyroxine than the restrictive/malabsorptive group. While these may be factors to consider when choosing the type of surgery for hypothyroid bariatric patients, further studies that are randomized, controlled, and multi-center with a higher population, should be performed.
